# Cell Line Techniques and Gene Editing Tools for Antibody Production: A Review

**DOI:** 10.3389/fphar.2018.00630

**Published:** 2018-06-12

**Authors:** Arun K. Dangi, Rajeshwari Sinha, Shailja Dwivedi, Sanjeev K. Gupta, Pratyoosh Shukla

**Affiliations:** ^1^Enzyme Technology and Protein Bioinformatics Laboratory, Department of Microbiology, Maharshi Dayanand University, Rohtak, India; ^2^Independent Researcher, New Delhi, India; ^3^Advanced Biotech Lab, Ipca Laboratories Limited, Mumbai, India

**Keywords:** cell line engineering, antibodies, CRISPR-Cas, gene editing, RNAi, ribozymes

## Abstract

The present day modern formulation practices for drugs are based on newer tools and techniques toward effective utilization. The methods of antibody formulations are to be revolutionized based on techniques of cell engineering and gene editing. In the present review, we have discussed innovations in cell engineering toward production of novel antibodies for therapeutic applications. Moreover, this review deciphers the use of RNAi, ribozyme engineering, CRISPR-Cas-based techniques for better strategies for antibody production. Overall, this review describes the multidisciplinary aspects of the production of therapeutic proteins that has gained more attention due to its increasing demand.

## Introduction

Antibodies, especially monoclonal antibodies (mAbs) and derived products like antibody-drug conjugates, Fc-fusion proteins and antibody fragments are widely known for their many diagnostic and therapeutic applications (Mahmuda et al., 2017). mAbs constitute the largest group of recombinant proteins that find applications in several diseases such as cancers, autoimmune diseases (rheumatoid arthritis), cardiovascular diseases, etc (Wang C. et al., 2017; Wang Y. et al., 2017; Marston et al., 2018). Thus, it is now a dominant product class within the biopharmaceutical market. As of early 2017, there were about 68 mAbs approved by the US FDA (Cai, 2017). The early approaches for generation of mAbs by hybridoma technology were limited by challenges such as hybridoma instability and the development of human anti-mouse antibodies, which led to their rejection by the patient’s immune system (Kunert and Reinhart, 2016; Wang et al., 2016). Furthermore, during the manufacturing process, several parameters such as reducing time to market, production in large quantities to meet the continuous increasing market demands, cost effectiveness, etc. are major issues which limits its worldwide utility (Li et al., 2010).

So far, the most preferred platforms for expression of any biopharmaceutical, including antibodies has been mammalian cells. This is largely attributed to their ability to produce large volumes of therapeutic antibodies and adaptability in large-scale production systems (Kunert and Reinhart, 2016). Most importantly, mammalian cells are able to carry out desired protein folding and post-translational modifications identical to those in human systems (Wells and Robinson, 2017). Thus, chances of their rejection by patients are low. Other than mammalian cells, alternative expression systems include *Pichia pastoris, Aspergillus niger*, and *Escherichia coli* (Shriver-Lake et al., 2017; Magana-Ortiz et al., 2018; Nylen and Chen, 2018. These have been explored for expression of smaller antibody fragments (Li et al., 2010; Kunert and Reinhart, 2016), but these expression platforms have limitations like formation of inclusion bodies, which greatly hamper the final product yield, inability for post-translational modifications and codon bias. Thus, mammalian cells are most preferable (Gupta and Shukla, 2017b).

**FIGURE 1:**
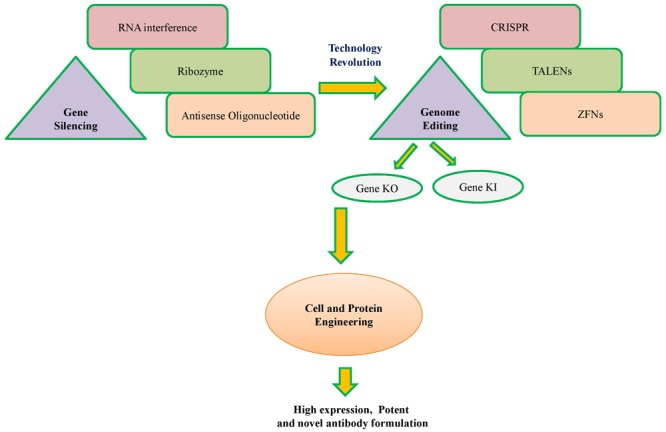
A schematic overview of technology revolution from conventional to modern cell engineering.

Further, advances in cell engineering make it more convenient to engineer the mammalian cells (Gupta et al., 2017b; Jazayeri et al., 2018). Engineering is basically used for regulating cellular apoptosis, cell cycle progression to avoid early cell death, control of cellular chaperones and ribozymes for proper post-translational modification of antibodies (Fischer et al., 2014; Vallée et al., 2014; Baek et al., 2017). Further, metabolic engineering can help balancing in-flux to utilized cellular energy toward antibody production. Recent discoveries and advances in gene editing nucleases like zinc finger nucleases (ZFNs), transcription activator-like effector nucleases (TALENs) and CRISPR/Cas systems (**Figure 1**) enable cell engineering more easy and cheaper (Gupta and Shukla, 2017a). These nucleases can alter the original genetic makeup of the cell by editing its genes toward achieving specific goals. Several studies have demonstrated the successful use of these nucleases in cell engineering (Kipniss et al., 2017; Moreno and Mali, 2017; Hashemi, 2018).

This review provides information about conventional and modern cell engineering techniques for more efficient antibody production. Overall, the review emphasizes different RNAi, ribozyme engineering and CRISPR-Cas-based techniques for better mAb production strategies.

## Cell Engineering Toward Production of Novel Antibodies for Therapeutic Applications

The most prominent mammalian host cell lines for recombinant mAb expression include the chinese hamster ovary (CHO), mouse myeloma derived NS0 and Sp2/0 cells, human embryonic kidney cells (HEK293), and human embryonic retinoblast-derived PER.C6 cells (Beck et al., 2008). CHO cells are believed to be the most used “workhorse” for today’s industrially produced recombinant products (Bahadori et al., 2017). One of the approaches toward obtaining enhanced production efficiency and improved quality of antibodies from mammalian cell lines is through engineering of the host cell (Wells and Robinson, 2017). Various types of cellular and genetic engineering techniques are therefore directed at modifying features specific to the host cells (Black et al., 2017; Yusufi et al., 2017). These may include approaches to manipulate growth of the cell, prevent death of the cell, promote post-translational modifications etc. This is achieved largely by regulation of apoptosis, metabolic engineering, engineering cells for growth at lower temperature, chaperone engineering and glyco-engineering, which are discussed below.

### Regulating Apoptosis in Cells

Apoptosis refers to programmed cell death induced during high stress conditions. Prevention of apoptosis in an antibodies expressing cell line will therefore increase cell viability, suppress cell death, extend cell culture life-span and increase productivity of target antibody product (Fischer et al., 2015a). In view of this, strategies to delay the onset of apoptosis, over-expressanti-apoptotic genes and down-regulate pro-apoptosis genes have been developed (Zustiak et al., 2014; Zhang et al., 2018). Over expression of anti-apoptotic genes such as *Mcl-1, 30Kc6, Bcl-2, Bcl-w, Bcl-xL, Aven, E1B-19K* and suppression of pro-apoptosis genes such as *Bax, Bok, Bak* in mammalian host cells have been documented to increase therapeutic protein production, including mAbs (Kim et al., 2012; Baek et al., 2017; Zhang et al., 2018). A recent study reported an 82% increase in production yield of antibody in CHO cells co-transfected with Bcl-x L, and 34% increase in CHO cells co-transfected with Mcl-1 (Zhang et al., 2018). Delaying the onset of apoptosis can be achieved by strategies such as periodic nutrient feeding, use of alternate carbon sources like galactose in place of glucose or use of adenosine (Costa et al., 2010). Inhibition of apoptosis by exosomes in CHO cells has been recently reported (Han and Rhee, 2018). Inhibition of the expression of caspases, which have an important role in the regulation of apoptosis, is another promising strategy (Costa et al., 2010; Zamaraev et al., 2017).

### Regulating Cell Cycle Progression

Inhibition of cell cycle progression is another approach which could lead to higher cell viability, density and productivity in mammalian cell cultures. Toward this, approaches such as inducible expression of cell-cycle regulating factors (p27 and p21^cip1^) and use of rapamycin have been shown to slow down the progression through cell cycle (Costa et al., 2010). Ibarra et al. (2003) showed that arresting the cell cycle in G1 phase of the NS0 6A1/4–9F myeloma cell line through inducible expression of p21^cip1^ increased IgG4 antibody production. Cell cycle arrest has also been achieved through inhibition of cyclin-dependent kinase (CDK) or over expression of CDK inhibitor. A small molecule, cell cycle inhibitor (CCI) was able to induce complete G0/G1 arrest in CHO cell cultures through selective inhibition of cyclin CDK 4/6 and led to improved specific productivity by twofold to threefold (Du et al., 2015). It has also been predicted that the mammalian target of rapamycin (mTOR) based engineering of mammalian cell lines can significantly influence production levels of therapeutic proteins in the long term (Dreesen and Fussenegger, 2011; Dadehbeigi and Dickson, 2015).

### Engineering of Chaperones

Chaperones and foldases have a critical role in mediating the folding of mAbs, produced by recombinant cell lines (Nishimiya et al., 2013). Engineering of chaperones has thus been found to influence production levels of antibodies through alterations in the translational capacity for the recombinant protein product (Pybus et al., 2014). Over expression of protein disulphide isomerase (PID), an enzyme that catalyzes the formation of disulphide bonds, moderately increased expression of mAb from CHO cells (Borth et al., 2005; Mohan et al., 2007). Another binding protein, BIP, associated with the folding pathway of secretory proteins, when over-expressed alone or in combination with PID, was reported to decrease mAb expression (Borth et al., 2005). Over-expression of X-box binding protein 1 (XBP1), another protein linked to the unfolded protein response, could be used as a strategy for enhancing recombinant protein production only when protein accumulation has surpassed the secretory capacity of the host cell (Ku et al., 2008).

### Post-translational Modifications

N-glycan structures on antibodies have been demonstrated to have a crucial role in its bioactivity and are linked to improvement in efficacy and safety of the mAb (Pancera et al., 2013). Common mechanisms of action of therapeutic mAbs are through the elicitation of antibody-dependant cell cytotoxicity (ADCC) and complement-dependent cytotoxicity (CDC). Any alterations to the N-glycan structure on antibodies can therefore influence such antibody-specific mechanisms. Antibodies produced in CHO cells are characterized by very low levels of bisecting-N-acetylglucosamine (GlcNAc) and high levels of core fucosylation. N-acetylglucosaminyltransferase III (GnT-III), when over-expressed has been found to increase the bisecting GlcNAc content to eventually improve ADCC (Davies et al., 2001; Popp et al., 2018). Decreasing or eliminating the fucose content on antibodies has also been known to enhance the ADCC activity. Non-fucosylated therapeutic antibodies have also been touted as the next generation of therapeutic antibodies (Mori et al., 2007). Toward this, one of the commonly used methods involves knocking out the fucosyltransferase gene (FUT 8) from CHO cells resulting in expression of non-fucosylated antibody molecules. The ADCC activity markedly improved in case of fucose-deficient mAbs (Shields et al., 2002; Yamane-Ohnuki et al., 2004; Chung et al., 2012).

### Metabolic Engineering

Accumulation of ammonia and lactate during recombinant CHO cell culture is common. This usually takes place due to the presence of glutamine and glucose in the culture medium and may cause adverse effects on the growing cells and the recombinant product secreted. Metabolic engineering has therefore been used to inhibit the accumulation of such toxic metabolic by-products. The over-expression of the glutamine synthetase (GS) gene in CHO cells enabled cells to grow in a glutamine-free medium, thus significantly reducing ammonia generation as a of byproduct in the culture (Zhang et al., 2006). Similarly, over-expression of enzymes such as ornithine transcarbamylase or carbamoyl phosphate synthetase I (enzymes associated with the urea cycle) has been found to bring down ammonia production within the culture medium (Park et al., 2000). In order to reduce lactic acid accumulation, various genetic modulation strategies that have been investigated include over-expression of pyruvate carboxylase (Henry and Durocher, 2011; Vallée et al., 2014; Gupta et al., 2017a) or disruption of pyruvate dehydrogenase kinases/lactate dehydrogenase A (LDH-A) (Kim and Lee, 2007; Zhou et al., 2011). LDH-A down regulation along with the GS system in the mAb-producing CHO cell line successfully reduced both ammonia and lactate levels in culture (Noh et al., 2017).

### Engineering Cells for Hypothermic Growth

Reducing the cell-culture temperature to improve recombinant protein yields in CHO cells has been well documented. Lowered temperature leads to arrest of growth of cells, prolonging cellular viability and increasing cellular size. Genetic engineering strategies have therefore been developed to improve the volume of recombinant protein production at low temperatures (Mohan et al., 2008; Costa et al., 2010). Expression of cold stress genes, such as cold-inducible RNA-binding protein (CIRP) is altered when mammalian cells are exposed to lower temperatures. Stable over-expression of CIRP at 37°C showed improvement in the productivity and yields of recombinant interferon-γ in the CHO cell line (Tan et al., 2008).

Cell line engineering therefore has great potential for improving CHO cell expression systems, particularly for therapeutic antibody production (**Table 1**). The benefits of cell line engineering are however not limited to antibody production only, but have also been explored in production of other biopharmaceutical products such as other recombinant proteins, vaccines, fusion proteins, growth factors etc. (Xiao et al., 2014; Genzel, 2015; Johari et al., 2015; Sandig et al., 2017). Cell engineering approaches have also been used to obtain a desired attribute or quality in the target product produced by a cell, reduce those host cell proteins which could act as impurity and adversely impact the final product during downstream processing, or identify of an optimal site in host cell genome in order to target a transgene to that location (Gadgil, 2017).

**Table 1:** Cell engineering approaches toward the production of novel antibodies for therapeutic applications.

Cell engineering approach	Strategies involved	Result	Reference
Regulation of apoptosis in cells	Delay onset of apoptosis	Limit cell-apoptosis	Costa et al., 2010
	Over-expression of anti-apoptotic genes		Costa et al., 2010; Baek et al., 2017; Zhang et al., 2018
	Inhibition or down-regulation of pro-apoptosis genes		Han and Rhee, 2018
Regulation of cell cycle progression	Inducible expression of cell-cycle regulating factors	Cell cycle arrest	Ibarra et al., 2003; Costa et al., 2010
	Inhibition of cyclin-dependent kinase (CDK) or over expression of CDK inhibitor		Du et al., 2015
	Use of mTOR- based engineering of mammalian cell lines	Slowed progression through cell cycle	Costa et al., 2010; Dreesen and Fussenegger, 2011; Dadehbeigi and Dickson, 2015
Engineering of chaperones and foldases	Over expression of protein disulphide isomerase	Increased formation of disulphide bonds in proteins	Borth et al., 2005; Mohan et al., 2007
Post-translational modifications	Knocking out the fucosyltransferase gene (FUT 8) from CHO cells	Enhanced ADCC activity	Shields et al., 2002; Yamane-Ohnuki et al., 2004; Chung et al., 2012
Metabolic engineering	Over-expression of glutamine synthetase gene in CHO cells	Reduction in ammonia generation as by-product within culture	Zhang et al., 2006
	Over-expression of ornithine transcarbamylase, carbamoyl phosphate synthetase I		Park et al., 2000
	Over-expression of pyruvate carboxylase	Reduction in lactic acid accumulation as by-product within culture	Henry and Durocher, 2011; Vallée et al., 2014; Gupta et al., 2017a
	Down-regulation of pyruvate dehydrogenase kinases/ lactate dehydrogenase A		Kim and Lee, 2007; Zhou et al., 2011
Engineering cells for hypothermic growth	Stable over-expression of cold stress genes, such as cold-inducible RNA-binding protein	Improvement in the productivity and yields of recombinant protein	Tan et al., 2008

## Ribozyme Engineering in Antibody Formulation

**FIGURE 2:**
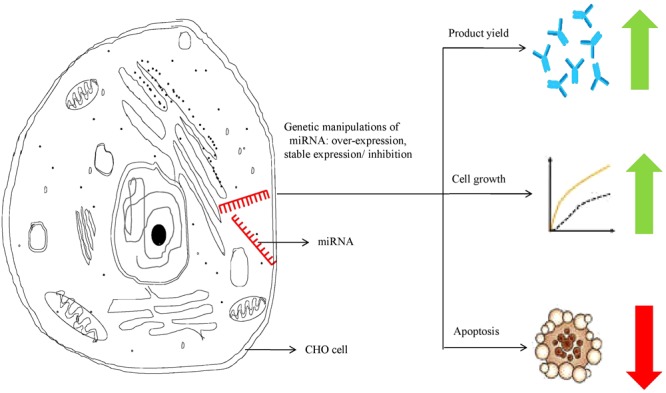
miRNA manipulation in CHO cells leading to improved therapeutic protein productivity.

RNAs not only serve to establish linkages between genetic information and proteins, but also play a key role as active regulators of gene expression. MicroRNAs (miRNAs) are one such class of RNA regulatory molecules. These are short non-coding RNAs, which are capable of regulating entire cellular pathways by post-transcriptionally modulating expression of numerous genes. miRNAs are associated with relevant cellular processes such as apoptosis, cell proliferation, or biosynthesis of proteins. Host cell engineering using miRNAs represents an important tool to overcome limitations in industrial cell-line development. miRNAs also provide better advantages over single-gene manipulation due to their ability to affect expression of multiple genes. Moreover, miRNAs, unlike cellular engineering (which relies largely on overexpression of regulatory proteins), are not translated into proteins. Thus, requirement of the translational machinery for miRNA over-expression is not there and there is no translational burden to the production cell line, even as the miRNAs regulate important cellular physiological processes (Druz et al., 2012; Hackl et al., 2012; Jadhav et al., 2013; Patel, 2017; Jazayeri et al., 2018). In recent years, available literature has focused on the identification of miRNAs to improve recombinant protein production (Fischer et al., 2014; Jadhav et al., 2014; Loh et al., 2014; Kelly et al., 2015), or enhanced cell growth (Barron et al., 2011; Druz et al., 2013; Sanchez et al., 2014). Manipulation of miRNA levels in CHO cells has been shown to improve product yield by increasing proliferation and specific productivity, resisting apoptosis and enhancing oxidative metabolism (**Figure 2**). Some of the recent studies on the effect of miRNA manipulations in CHO cells have been summarized in **Table 2** below.

**Table 2:** Recent studies on the effects of miRNA manipulations in CHO cells.

miRNA	Fate in host cell (CHO cells)	Key observations	Reference
cgr-miR-7	Over expression	Increase in cell’s specific productivity	Barron et al., 2011
miR-17	Over expression	Increased cell proliferation; no negative impact on cell’s specific productivity	Jadhav et al., 2012
miR-557, miR-1287	Stable expression	Enhanced viable cell density and specific productivity of therapeutic IgG1	Strotbek et al., 2013
Mmu-miR-466h-5p	Stable inhibition	Improved resistance to apoptosis; improved protein production	Druz et al., 2013
miR-30 family	Stable over expression	Improvement in cell’s bioprocess performance	Fischer et al., 2014
miR-17	Stable over expression	Enhanced cellular growth; cell’s specific productivity increased by two fold	Jadhav et al., 2014
miR-17, miR-1b, miR-92a	Over expression	Enhanced cellular productivity	Loh et al., 2014
miR-2861		Enhanced cellular productivity	Fischer et al., 2015b
miR-557	Stable expression	Significant increase in yield of final product	Fischer et al., 2017

The **Table 2** shows that miRNAs have largely been subjected to stability or over-expression in CHO cell lines, leading to improved protein productivity. For example, CHO cells, which have been engineered to express miR-557, a pro-productive miRNA, have shown an increase in product yield without compromising product quality (Fischer et al., 2017). Expression of miR-30 and miR17 in CHO cells have also led to enhanced specific productivity (Fischer et al., 2014; Jadhav et al., 2014). A study assessing the effect of over-expression of miR-7 on the proteome of CHO cells showed that the cell’s productivity increased with increasing levels of miR-7, albeit at the cost of cell growth (Meleady et al., 2012). Sanchez et al. (2014) have reported an increase in protein production by CHO cells, when miR-7 activity was disrupted using sponge decoy vectors. On the contrary, stable inhibition of mmu-miR-466h-5p in CHO cells has been found to increase resistance to apoptosis and also improve protein production (Druz et al., 2013). Such stable manipulations of miRNA expression can also enable CHO cells to reach a higher cell viability and cell density in comparison to their controls.

miRNA-based cell line engineering provides an opportunity for access to numerous sophisticated therapeutic proteins, including antibodies. While this approach has not yet found a way into routine industrial production processes, there lies a significant potential for their future application in industrial manufacturing cell lines, ultimately contributing to successful biopharmaceutical drug development. While miRNAs are deemed to have multiple applications in medicine and biotechnology, a better understanding of their mechanism at the molecular level is needed to harness their potential toward the development of industrially relevant therapeutic protein-producing cell factories.

## Advanced Gene Editing Tools

With the discovery and emergence of modern gene-editing tools, manipulation of production hosts has become relatively easy. It is now possible to maneuver the genome of industrial yeast and mammalian host cells, thereby allowing the development of potential and cost-effective recombinant therapeutic proteins. While previously developed gene editing tools such as TALENs and ZFNs continue to remain useful, the emergence of another new technology, the CRISPR-Cas system is opening new avenues in gene editing opportunities as described below (Sander and Joung, 2014).

### Zinc Finger Nucleases (ZFNs)

Most commonly and primitive endonucleases are ZFNs which are artificially designed restriction enzymes (Yang et al., 2017). Zinc finger proteins (ZFPs) derived from eukaryotic transcription factors, which act as a DNA binding domain of ZFN and the nucleotide chopper domain Folk 1 is derived from *Flavobacterium okeanokoites* (De Souza, 2012). The ZFPs are composed of 30 amino acids which creates two anti-parallel β-sheets opposite an alpha-helix (De Souza, 2012). Each cleavage domain is attached with three to six ZFPs, which are specially designed for a target site. Usually, ZFPs have 18 base pair specificity and DNA-binding domains have 9 base pair specificity, which makes them robust and very precise for target-specific gene editing (Sander et al., 2011b). Till date, the ZFNs have been successfully utilized for HDR-mediated gene knock-in (KI) and non-homologous end joining (NHEJ) based Knock-out (KO) approaches to many prokaryotic and eukaryotic targets (Carroll, 2011). For example CHO cells are modified by KI of GS and dihydrofolate reductase (DHFR) genes, which makes them suitable for consistent protein production in therapeutic biopharmaceutical industries. Even though, after successful experiments, ZFNs are not considered for industrial use now a days due to certain restrictions like: A. All possible nucleotide genomic sequences can’t be targeted, B. Specificity of sequence can be interrupted by neighboring protein domains (Sander et al., 2011b). Apart from these, designing of folks and ZFPs are very complicated and costly which makes it less preferred.

### Transcription Activator-Like Effector Nucleases (TALENs)

Transcription activator-like effector nucleases are a moderately innovative gene-editing tool. TAL proteins are the major part of TALENs, which are secreted from the pathogenic bacteria *Xanthomonas* (Li et al., 2012). TALENs have tandem repeats of 34 amino acids with specific recognition and binding efficiency. The TAL has the capability of recognizing a single nucleotide, which is very specific and not influenced by other domains present around it. The TALENs based gene editing tool is relatively more effective and frequently used for knock-in and knock-out events. Similarly, like ZFNs, TALENs also have two-site two protein domains, which create cuts and second TAL repeats, which helps protein domains to find a specific site for binding and making a notch (Christian et al., 2010). TALENs leave sticky ends to the DNA, which minimize the off-target results (Aryan et al., 2013). Efficiency of targeted KO with TALENs is 30 to 100% while KIs range from 1 to 10%. Many animals like zebrafish, chickens, frogs, rats, and mammalian cells are genetically modified through TALENs (Sander et al., 2011a; Aryan et al., 2013; Kondo et al., 2014).

### CRISPR-Cas

CRISPR-Cas is based on the natural defense mechanism in bacteria, which they use for the control of pathogens. Cas9 endonuclease from *Streptococcus pyogenes* has been one of the most studied with respect to CRISPR-Cas systems (Ran et al., 2015). Cas9 endonuclease binds to a complex of CRISPR RNA (crRNA) and another transactivating crRNA (tracrRNA). The crRNA guides the Cas9 endonuclease activity to cut both strands of target DNA at particular sequences (Briner et al., 2014). The ability of CRISPR-Cas 9 to target the Cas9 endonuclease to specific genomic loci, enabling DNA cleavage at specific sites has allowed it to being considered as an adept tool in gene editing (Doudna and Charpentier, 2014). CRISPR-Cas-mediated gene repair, disruption, insertion or deletion is thus finding applications in several areas of biomedical research, medicine, agriculture and biotechnology (Hwang et al., 2013). Recent studies utilizing CRISPR-Cas-based systems for genetic manipulations in animals, fish and plant models have been summarized in **Table 3**.

**Table 3:** CRIPSR-Cas based gene editing techniques used for manipulation of various organisms.

Model organism	CRISPR-Cas based gene manipulations	Observations/Remarks	Reference
Zebrafish	Genetic modifications in embryo of zebrafish	Similar efficiency to ZFN and TALENs	Hwang et al., 2013
Zebrafish	Insertion of *CreER^T2^* transgene at *otx2* gene locus	*otx2*:*CreER ^T2^* transgenic fish developed; valuable tool for future studies	Kesavan et al., 2018
Mouse	Simultaneous disruption of five genes (*Tet1, 2, 3, Sry, Uty -* 8 alleles) in embryonic stem cells of the mouse	High efficiency observed	Wang et al., 2013
Mouse	Editing of specific regions of the Duchenne muscular dystrophy gene using AAV vectors	Expression of Cas9 enables direct editing of the mutation, deletion of multiple exons or gene correction	Bengtsson et al., 2017
mdx Mouse	Correction of the dystrophin gene (*Dmd*) mutation	2-100% correction of the *Dmd* gene achieved	Long et al., 2014
Mouse	Deletion of the GAA repeats from frataxin gene	Restoration of frataxin gene activity and protein level	Ouellet et al., 2017
Mouse	Mutation of *Pten* and *p53* in mouse liver	Development of models for liver cancer	Xue et al., 2014
Mouse	*Dip2a* gene deletion and β-galactosidase (lacz) reporter gene insertion	Development of mouse models and studies on gene function	Zhang L. et al., 2015
Mouse	*EGFP* transgene or *Crygc* gene mutation in Spermatogonial Stem Cells (SSCs)	Spermatogenesis observed in mutated SSCs after being transplanted into seminiferous tubules of infertile mouse testes.	Wu et al., 2015
Duchenne muscular dystrophy (DMD) patient-derived induced pluripotent stem cells (iPSCs)	Correction of dystrophin gene	Restoration of the dystrophin protein in patient-derived iPSCs	Li et al., 2015
Human pluripotent stem cells (hPSCs)	Development of an episomal vector-based CRISPR/Cas9 system which enables generation of up to 100% Insertion/Deletion rates	Highly efficient gene knockout in hPSCs	Xie et al., 2017
*Indica* rice line IR58025B	Deleted DNA fragments in the gene *Dense And Erect Panicle1* (*DEP1*)	Relatively high frequency	Wang Y. et al., 2017
Tomato plants	Somatic mutations in *SlIAA9* gene	Morphological changes in leaf shape and seedless fruit observed in mutants	Ueta et al., 2017
Maize plants	Gene sequence *ARGOS8* edited	Increased yield of maize grain under stress conditions such as drought	Shi et al., 2017
Oilseed crop *Camelina sativa*	Mutagenesis of 3 delta 12 desaturase (*FAD2*) genes	Reduction in polyunsaturated fatty acid levels; increased oleic acid accumulation in oil	Morineau et al., 2017

The CRISPR-Cas system has not only enabled gene editing but also has applications across several areas such as gene therapy, development of tissue and animal disease models, drug discovery, deciphering plant disease resistance, transcription regulation, genome imaging, and epigenetic modification (Xiong et al., 2016). CRISPR-Cas-based gene editing has been used for development of animal models, that mimic human diseases. Such models provide an opportunity to predict possible outcomes of clinical trials and simultaneously verify the safety and accuracy of drugs against the particular disease. Recently, CRISPR-Cas9 technology has been used to establish large animal models that can mimic human neurodegenerative diseases for enhanced understanding of pathogenesis of such diseases (Tu et al., 2015). The CRISPR-Cas9 system has also been used for successful correction of diseases or disease-causing mutations in animal models (Wu et al., 2013; Long et al., 2014). In a recent report by Cai et al. (2016) it was summarized how the CRISPR-Cas9-based genome editing technology is opening doors for treatment of diverse human diseases. The technology has recently been applied for the study or treatment of human diseases such as muscular dystrophy, hemophilia, thalassemia, cystic fibrosis etc. The CRISPR-Cas system has been used for correction of the dystrophic gene in Duchenne muscular dystrophy (DMD) patient-derived induced pluripotent stem cells and restoration of the dystrophin protein in the cells (Li et al., 2015). The tool has been used for immunology-based applications, which may lead to designing of treatment regimens of diseases like HIV-AIDs (Hu et al., 2014; Liao et al., 2015). It has also been exploited for its potential to KI or KO specific genes in model organisms for studying genetic diseases (Platt et al., 2014; Kang et al., 2015).

The CRISPR–Cas system also has a big part to play in revolutionizing the role that gene editing plays in drug discovery. However, CRISPR–Cas-facilitated drug discovery is presently largely limited to basic research (Golkar et al., 2016; Luo, 2016). CRISPR’s potential is believed to span each stage of the drug discovery process and will predictably affect the next generation drugs by accelerating drug target identification and validation, discovery of biomarkers, and development of novel therapies (Fellmann et al., 2017; Lu et al., 2017). Alternatively, CRISPR-Cas can also be used in order to generate disease genotypes or phenotypes for use in drug discovery. CRISPR/Cas-based gene editing tools can also play a potential role in antiviral drug development (Chen et al., 2018). Mutations in Hepatitis B virus (HBV) DNA, brought about by the CRISPR-Cas system, inhibit replication and expression of the HBV and may constitute a new therapeutic strategy for HBV infection (Zhen et al., 2015). Similarly, human primary CD4^+^ T cells have shown HIV-1 resistance owing to disruption of the human CXCR4 gene by CRISPR/Cas9-mediated genome editing (Hou et al., 2015). In addition to the above, CRISPR-Cas system has been implicated in slowing down the spread of antibiotic resistance genes. The system has the ability to limit major routes of horizontal gene transfer and even destroy plasmids thereby restricting the spread of drug resistance (Golkar et al., 2016). This ability of the CRISPR/Cas system also holds potential to be exploited in clinical settings.

In addition to their role in designing disease treatment regimens or drug discovery, advanced gene editing technologies such as CRISPR-Cas9 also hold significant potential for the future in that they will provide new opportunities to stably engineer host cells for antibody production. This has been explored in some of the recently published research. CRISPR/Cas9 based genome editing has been used for knockout of GS-encoding gene resulting in improved recombinant protein production in CHO cells (Grav et al., 2017). The inactivation of GDP-fucose transporter gene in CHO cells by ZFNs, TALENs and CRISPR-Cas9 has been reported to lead to production of fucose-free antibodies (Chan et al., 2016).

However, TALENs and ZFNs are starting to prove their worth in human gene targeting and editing. The biotech company Sangamo Biosciences (California, United States) has stepped forward its ZFN in *in vivo* gene editing research into the clinic, and the US Food and Drug Administration (US-FDA) has permitted a HIV program in somatic cells (non inherited cells), which has cured more than 80 HIV-infected patients so far (Xu et al., 2017). Meanwhile, TALENs are recently used in the clinic to engineer immune cells to help fight leukemia in a young girl (Le, 2015). Despite these successes, many researchers found CRISPR as a less onerous and more efficient tool as compared to other two gene editing tools described above. The cost savings of the new method is also substantial. Thus, genome editing approaches therefore present a tremendous scope for being exploited for the production of novel antibodies with varying applications.

## Conclusion and Future Prospective

Although, mammalian cells are employed for production of more effective mAbs for diagnostics and treatment of many severe and life-threatening diseases, certain limitations prevent their dominancy (Zhu, 2012; Ye et al., 2016). Advances in cell engineering approaches at various stages, therefore enables cells to be used as a sole and improved platform for production of complex natured mAbs. Some of the exiting cell engineering approaches have been discussed in this review. These include by regulation of apoptosis, metabolic engineering, and engineering cells for growth at lower temperature, chaperone engineering and glyco-engineering. Engineering of cells toward regulation of the apoptosis or cell-cycle progression can result in longer cell viability and higher cellular productivity. Specific cellular chaperones and foldases can be engineered for proper folding of nascent polypeptide chains to an antibody (Zhang et al., 2018). Other approaches are targeted for reduction of metabolic by-product accumulation, enhancing cellular productivity at lower cell-culture temperatures or obtaining a desired attribute or quality in the target protein product. Additionally, cell line engineering has also contributed to the production of other biopharmaceutical products, reduction of impure host cell proteins, etc. Host-cell engineering using micro-RNAs also represent a recent and important strategy that can help improve recombinant therapeutic protein production. There lies immense scope for exploring miRNA based cell-line engineering in industrial protein production processes. Discovery of novel gene editing tools including ZFNs, TALENs and CRISPR/Cas have made the cell engineering more convenient to achieve specific goals (Gupta and Shukla, 2017a). Among these three, the CRISPR/Cas9 system is now gaining its own importance in curing human genetic diseases as well as host engineering for potential biopharmaceutical production (Ran et al., 2015). With the advent of the CRISPR/Cas9 tool, it is now possible to target the human genome at specific sites to cure various life threatening genetic diseases such as HIV, Leukemia, Sickle cell anemia etc. This tool has also been potentially used for human gene therapy with success. As CRISPR/Cas9 technology is still in a nascent phase, certain limitations need to be overcome. Off-target effects are the major ones which may cause undesirable editing or mutation in the host cell genome (Zhang X.H. et al., 2015). The designing of sgRNA should be very specific for a particular gene; otherwise, it can edit off-targets (non-specific), which may cause unwanted editing or mutation in the host genome (Doench et al., 2016). Availability of the PAM sequence is also very important because the Cas9 protein recognizes only the PAM (NGG) sequence at the time of binding to insure the spacer’s attachment. If any mutation occurs in the PAM sequence during *in vitro* processing, the cleavage of the target DNA will be restricted. Therefore, specificity is a must for the locus identification as well as the designing of the sgRNA. It can be predicted that, the CRISPR/Cas9 platform can one day partially replace the drugs, because scientists have developed this tool for treating critical diseases such as cancers, HIV leukemia etc. (Doudna and Charpentier, 2014). However, the CRISPR modifies a gene, the change is permanent. The other limitation is availability of CRISPR, since it comes from bacteria; if it stays in the body for too long, it might trigger a deleterious immune response (Seed et al., 2013). Many ethical issues have already been raised against germ-line cell editing with CRISPR, because it can affect evolution and can raise the ethical and justice issues. It is essential to have strong ethics for the use of CRISPR or any other gene editing tool. These tools have the capability of KI or KO on specific genes at loci for metabolic engineering as well as improved protein production and product quality, which in turn can be beneficial in reducing drug production costs and increased affordability for a large population.

## Author Contributions

All authors listed have made a substantial, direct and intellectual contribution to the work, and approved it for publication.

## Conflict of Interest Statement

SG and SD were employed by company Ipca Laboratories (India) Mumbai, India. The remaining authors declare that the research was conducted in the absence of any commercial or financial relationships that could be construed as a potential conflict of interest.
